# Searching for a better formulation to enhance muscle bioenergetics: A randomized controlled trial of creatine nitrate plus creatinine *vs.* creatine nitrate *vs.* creatine monohydrate in healthy men

**DOI:** 10.1002/fsn3.1237

**Published:** 2019-10-03

**Authors:** Sergej M. Ostojic, Valdemar Stajer, Milan Vranes, Jelena Ostojic

**Affiliations:** ^1^ Applied Bioenergetics Lab Faculty of Sport and PE University of Novi Sad Novi Sad Serbia; ^2^ Faculty of Health Sciences University of Pecs Pecs Hungary; ^3^ Faculty of Sciences University of Novi Sad Novi Sad Serbia; ^4^ Faculty of Health Sciences University of Southern Denmark Odense Denmark

**Keywords:** creatine monohydrate, creatine nitrate, supplementation, the area under the curve, tissue uptake

## Abstract

A novel creatine blend (creatine nitrate mixed with creatinine, CN‐CRN) has been anecdotally suggested to be superior to traditional creatine formulations for bioavailability and performance. However, does CN‐CRN supremely affects creatine levels in the blood and skeletal muscle of healthy humans remain currently unknown. This randomized, controlled, double‐blind, crossover trial evaluated the acute effects of single‐dose CN‐CRN on serum creatine levels, and 5‐days intervention with CN‐CRN on skeletal muscle creatine and safety biomarkers in healthy men. Ten healthy young men (23.6 ± 2.9 years) were allocated to receive either CN‐CRN (3 grams of creatine nitrate mixed with 3 grams of creatinine), pure creatine nitrate (3 grams, CN), or regular creatine monohydrate (3 grams, CRM) by oral administration. We found that CN‐CRN resulted in a more powerful rise in serum creatine levels comparing to either CN or CRM after a single‐dose intervention, as evaluated with the area under the concentration–time curve calculation (701.1 ± 62.1 (µmol/L) × min versus 622.7 ± 62.9 (µmol/L) × min versus 466.3 ± 47.9 (µmol/L) × min; *p* < .001). The peak serum creatine levels at 60‐min sampling interval were significantly higher in CN‐CRN group (183.7 ± 15.5 µmol/L), as compared to CN group (163.8 ± 12.9 µmol/L) and CRM group (118.6 ± 12.9 µmol/L) (*p* < .001). This was accompanied by a significantly superior increase in muscle creatine levels after CN‐CRN administration at 5‐days follow‐up, as compared to CN and CRM, respectively (9.6% versus 8.0% versus 2.1%; *p* = .01). While 2 out of 10 participants were found to be nonresponsive to CN intervention (20.0%) (e.g., no amplification in muscle creatine levels found at 5‐days follow‐up), and 3 participants out of 10 were nonresponsive in CRM trial (30%), no nonresponders were found after CN‐CRN administration, with individual upswing in total muscle creatine varied in this group from 2.0% (lowest increment) to 16.8% (highest increment). Supplemental CN‐CRN significantly decreased estimated glomerular filtration rate (eGFR) at 5‐days follow‐up, as compared to other interventions (*p* = .004), with the average reduction was 14.8 ± 7.7% (95% confidence interval; from 9.3 to 20.3). Nevertheless, no single participant experienced a clinically relevant reduction in eGFR (< 60 ml/min/1.73 m^2^) throughout the course of the trial. Liver enzymes remained in reference ranges throughout the study, with no participant experienced high liver blood tests (e.g., AST > 40 units per L or ALT >56 units per L). Besides, no participant reported any major side effects during the trial, while the odors of CN‐CRN and CN formulations were considered somewhat unpleasant in 8 out of 10 participants (80.0%). Our results suggest that CN‐CRN is a preferred and relatively safe alternative to traditional creatine formulations for improved creatine bioavailability in the blood and skeletal muscle after single‐dose and 5‐days interventions.

## 1 INTRODUCTION

Creatine has become one of the most popular dietary supplements in the world, with creatine‐containing dietary supplements make up a large portion of the estimated $2.7 billion in annual sales of sports nutrition supplements in the United States alone (Jagim et al., 2018). Its effectiveness to enhance tissue bioenergetics remains a pillar of creatine power in both athletic and clinical environment, with creatine monohydrate (CRM) has been recognized as a most prevalent chemical form of creatine in the market (Kreider et al., 2017). Although effective in many therapeutic conditions, it appears that CRM might have some limitations concerning its poor solubility in water and inadequate delivery to specific energy‐demanding tissues and/or pathologies, including skeletal muscle‐ and brain‐related conditions (Bender & Klopstock, 2016). While CRM is readily absorbed from the gut into the circulation (Harris et al., 2002), a degree of creatine‐to‐creatinine conversion by nonenzymatic dehydration might affect its cellular uptake, mainly controlled by a saturable creatine transporter (CRT1 or SLC6A8) (Ostojic, 2017; Wyss & Kaddurah‐Daouk, 2000). Therefore, various formulations of this critical compound of energy metabolism are continuously developed to improve cellular uptake and performance of creatine in health and disease (Andres et al., 2017). A novel creatine formulation (creatine nitrate mixed with creatinine, CN‐CRN) has been anecdotally reported to be superior to CRM or creatine nitrate (CN) for bioavailability and performance. However, no human study so far evaluated its potency to affect blood and tissue creatine kinetics after oral administration, neither CN‐CRN safety outcomes for liver and kidney function tests. In the present preliminary study, we evaluated the pharmacokinetics of single‐dose CN‐CRN intervention for serum creatine and creatinine, and the effects of 5‐days intervention with CN‐CRN on skeletal muscle creatine levels, clinical chemistry, and subjective side effects in young healthy men. If proven effective and safe, this first‐in‐human phase I trial in healthy participants could pave the way for further studies with CN‐CRN in clinical environment.

## 2 MATERIALS AND METHODS

### 2.1 Participants

Ten healthy young men (age 23.6 ± 2.9 years, body mass index 23.8 ± 1.5 kg/m^2^) were recruited and signed informed consent to voluntarily participate in this double‐blind, crossover, randomized controlled trial. All experimental procedures were approved by the local IRB (Ethical Approval No. 102‐TL/2018), with the study conducted under the Declaration of Helsinki. All participants had no history of creatine supplementation (or other dietary supplements) within the 4 weeks before the study commenced, and no acute or chronic diseases, as evaluated by the preparticipation health check and blood profiles. All participants were required not to change activity patterns and diet during the study. Besides, participants were not engaged in any exhaustive exercise 24 hr before the study commenced.

### 2.2 Experimental protocol

**Figure 1:**
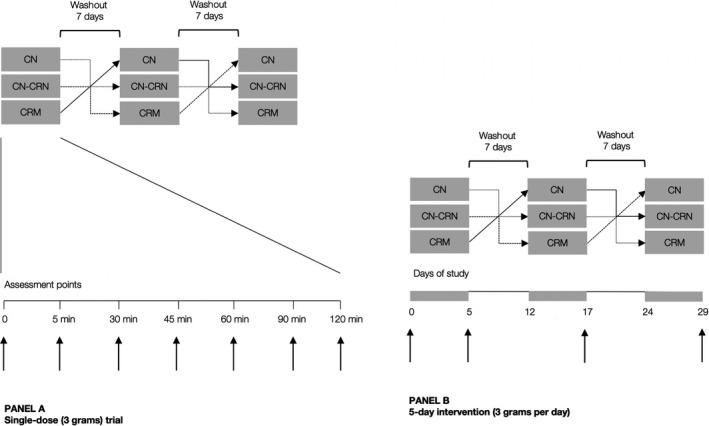
Study protocol for a single‐dose trial (Panel A) and 5‐day intervention (Panel B). The vertical arrows indicate sampling intervals for serum creatine and creatinine (Panel A) and muscle creatine and clinical biochemistry (Panel B)

The participants were assigned to receive either CN‐CRN (3 grams of creatine nitrate and 3 grams of creatinine), 3 grams of CN, or 3 grams of CRM by oral administration in a single‐dose pharmacokinetics experiment, and for 5‐days intervention (Figure 1). The amount of creatine used (3 g/day) was chosen as a minimal dose that gives the desired effect (e.g., an increase in muscle creatine) (Hickner, Dyck, Sklar, Hatley, & Byrd, 2010). Since a previous study has shown CN to be superior to CRM in terms of efficacy (Galvan et al., 2016), we added CRN to the CN dose only, while CRM was included as a control dose for a comparison. A washout period of 7 days was employed between all trials, to prevent the residual or carryover effects of treatments across study periods. For a single‐dose experiment, a researcher has prepared the intervention by dissolving the powder in 150 ml of lukewarm water and directly controlled the intake of the intervention. For a 5‐days intervention, the participants received five powder sachets (one per day) containing an intervention and were advised to stir the content of the sachet into 150 ml of lukewarm water until the powder has dissolved, and drink it ~ 30 min before breakfast. Creatine nitrate was supplied by ThermoLife International LLC (Phoenix, AZ), while creatine monohydrate (CreaPure^®^) was purchased from a retailer store (ATP Sport, Belgrade). Creatinine anhydrous has been produced from creatine monohydrate in an autoclave (Memmert UF 55) at 160 degrees centigrade for 72 hr, with creatinine purity >99% in a final powder, as determined by NMR spectroscopy (Bruker Avance III 400). Clinical assessments for both experiments were carried out between 08:00 and 12:00 after an overnight fast of 12 hr. Venous blood samples were drawn at each time point (seven points in total) for a single‐dose experiment, centrifuged within the next 10 min at 3,000 g, with serum separated, and immediately frozen at −80°C. Samples were analyzed for creatine and creatinine by modified LC‐MS/MS (1,200 Series LC System; Agilent Technologies Inc.) after the completion of the study. To control for hydration, all participants were restricted from water ingestion between baseline and 120‐min postadministration assessment. For a 5‐day experiment, venous blood samples were drawn at baseline and follow‐up, centrifuged within the next 10 min at 3,000 g, with serum separated, and immediately analyzed for liver enzymes by an automated analyzer (Randox Laboratories Ltd.). Estimated glomerular filtration rate (eGFR) was calculated by the abbreviated MDRD equation (Stevens et al., 2007).

Proton magnetic resonance spectroscopy (MRS) was performed on 1.5 Tesla Avanto scanner (Siemens) using a flexible phased array body‐matrix coil, with metabolite spectra in the right vastus medialis muscle processed with AMARES. A standardized volume of interest (20 × 20 × 20 mm^3^) was positioned within the right vastus medialis muscle with attention to avoid blood vessels, subcutaneous and other fat, and osseous structures. After local shimming and gradient adjustments, data were obtained with 256 data points and 32 non‐water‐suppressed scans using a point‐resolved spectroscopic sequence to acquire a single volume (TR/TE, 2000/135 ms). We obtained 12 datasets in each individual yielding a total of 120 spectra in this study. In vivo metabolite concentrations were calculated using the muscle water signal as an internal intensity reference (Wang, Salibi, Fayad, & Barker, 2014). The spectra were fitted in the time domain using a nonlinear least‐squares algorithm in the Java‐based magnetic resonance user interface (jMRUI) software package. The peak integral values of the total creatine (creatine plus phosphocreatine) signal at 3.0 ppm and the nonsuppressed water signal at 4.7 ppm were quantified using curve fitting to Gaussian lines. The signals were corrected for T1 and T2 relaxation using the T1 and T2 relaxation times as previously described (Varghese et al., 2015). Finally, possible adverse events of the intervention (e.g., muscle cramps, bloating, and diarrhea) were evaluated via open‐ended questionnaires administered during the trial.

### 2.3 Statistical analyses

A total number of subjects (*n* = 10) were calculated with effects size set at 0.9, two‐tail alpha level 0.05, and study power 0.80, with primary outcome was the change in total creatine levels in the serum assessed at baseline and 120 min postadministration. Two‐way ANOVA design for repeated measures (treatment vs. time) was used to establish if any significant differences existed between three interventions ingested during the experiment. Where significant differences were found, the Tukey post hoc test was employed to identify the differences. The area under the concentration–time curve (AUC) for serum creatine and creatinine was calculated using the linear‐log trapezoidal method, with AUCs compared with paired *t* test. The significance level was set at *p* < .05. Data were analyzed using the SPSS program (SPSS Inc.).

## 3 RESULTS

### 3.1 Single‐dose experiment

**Figure 2:**
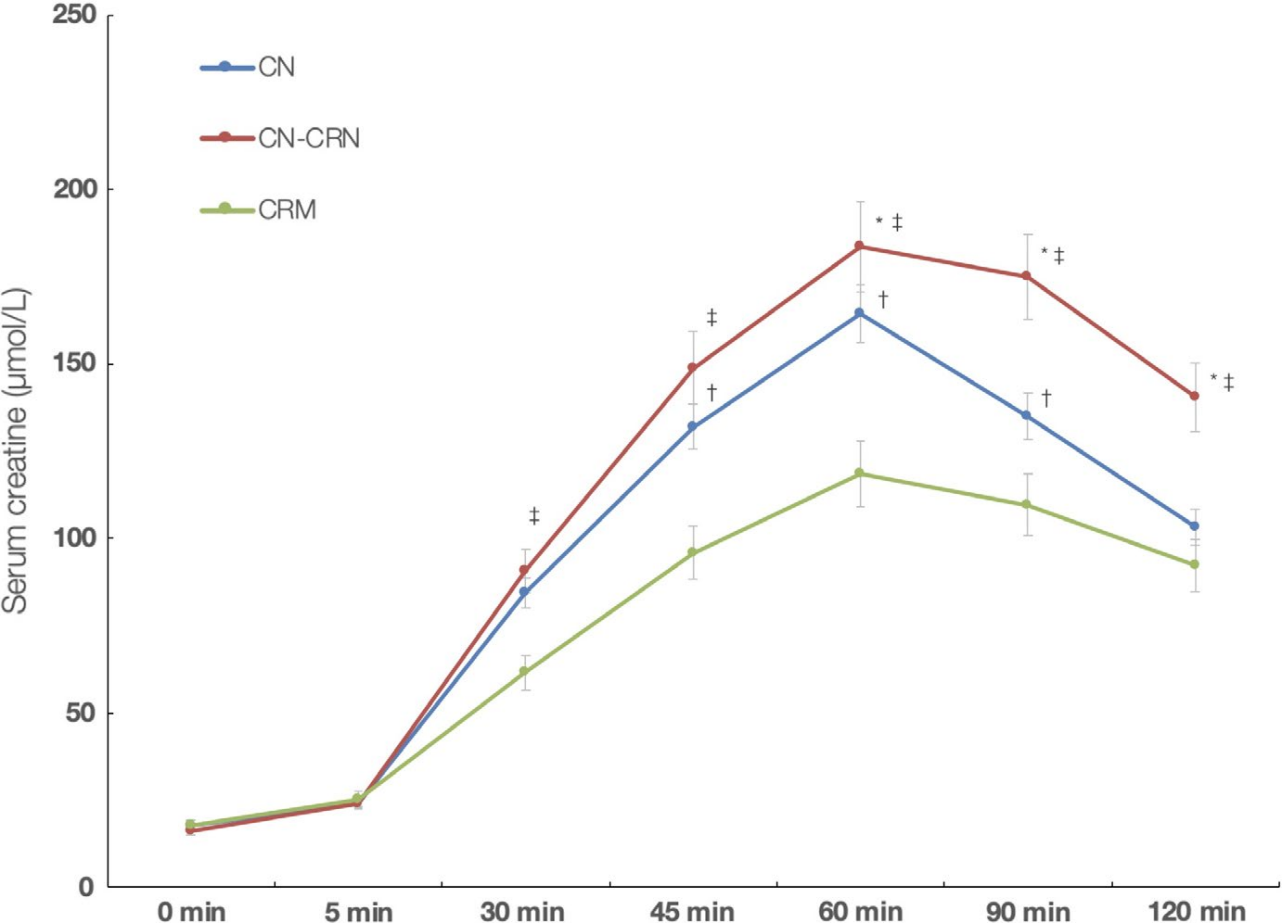
Changes in serum creatine levels during the course of the single‐dose study. *Indicates significant difference CN versus CN‐CRN at *p* < .05; †Indicates significant difference CN versus CRM at *p* < .05; and ‡Indicates significant difference CN‐CRN versus CRM at *p* < .05

All participants completed the trial, with no single participant reported any side effect of either intervention, although the odors of CN‐CRN and CN formulations were considered unpleasant by 8 of 10 men (80%). The compliance for all three groups was 100%. Changes in serum creatine levels during the single‐dose experiment are depicted in Figure 2. All three interventions induced a sharp rise in serum creatine concentrations starting at 5 min postintervention, with peak levels achieved after 60 min postintervention in all three groups, followed by a moderate reduction in creatine levels by the end of the experiment. The average creatine concentrations at 60‐min sampling interval were significantly higher in CN‐CRN group (183.7 ± 15.5 µmol/L), as compared to CN group (163.8 ± 12.9 µmol/L) and CRM group (118.6 ± 12.9 µmol/L) (*p* < .001). In addition, CN‐CRN resulted in a more advanced body exposure to creatine comparing to either CN or CRM after single‐dose intervention, as evaluated with AUC calculation (701.1 ± 62.1 (µmol/L) × min versus 622.7 ± 62.9 (µmol/L) × min versus 466.3 ± 47.9 (µmol/L) × min; *p* < .001).

**Figure 3:**
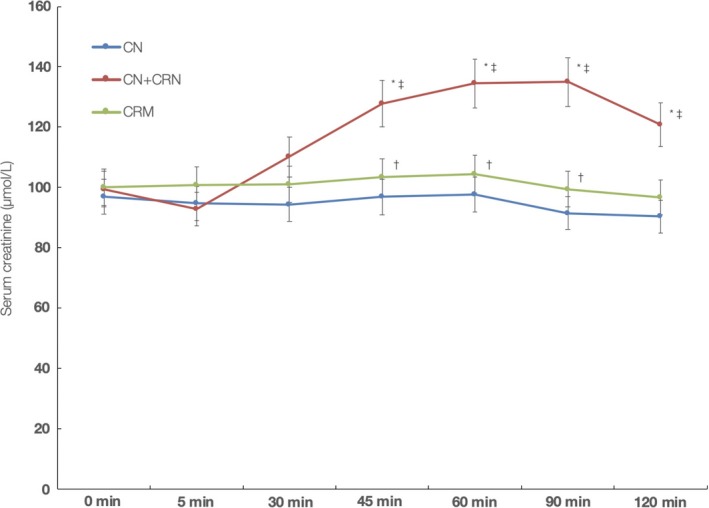
Changes in serum creatinine levels during the course of the single‐dose study. *Indicates significant difference CN versus CN‐CRN at *p* < .05; †Indicates significant difference CN versus CRM at *p* < .05; and ‡Indicates significant difference CN‐CRN versus CRM at *p* < .05

Serum creatinine remained essentially unchanged during the study in CN and CRM groups (Figure 3). However, serum creatinine increased significantly in CN‐CRN group, with peak values noted after 90 min postadministration (135.0 ± 17.1 µmol/L), an increase of 21.5 ± 13.0% from the baseline values. AUC analysis for creatinine revealed a significant difference between CN‐CRN, CN, and CRM groups, respectively (710.4 ± 49.2 (µmol/L) × min versus 568.9 ± 24.7 (µmol/L) × min versus 607.5 ± 37.7 (µmol/L) × min; *p* < .001), respectively.

### 3.2 5‐day intervention

All participants completed the 5‐day intervention, and the compliance with the regimen was 100% for CN group, 98.0% for CN‐CRN group, and 100% for CRM group, with unused powder sachets used to determine participants' compliance. A single participant (age 30, weight 69.5 kg) reported a single episode of irregular nondiarrheal bowel movements each day throughout the intervention with CN, and also for the first 2 days after CN‐CRN intervention, with the episode typically appeared ~2 hr after an administration. Another participant (age 19, weight 78.7 kg) reported excessive sleepiness in the course of CN intervention. Finally, a participant (age 22, weight 80.0 kg) reported seldom episodes of bloating throughout CRM intervention. Other participants reported no side effects of either intervention. The effects of different interventions on selected safety biomarkers are depicted in Table 1. No significant differences were observed between treatment groups in alanine aminotransferase (ALT) and aspartate aminotransferase (AST) among participants receiving CN, CN‐CRN, or CRM (*p* > .05). Liver enzymes remained in reference ranges throughout the study, with no participant experienced high liver blood tests (e.g., AST > 40 units per L and/or ALT > 56 units per L). Supplemental CN‐CRN significantly decreased eGFR at 5‐day follow‐up, as compared to other interventions (*p* = .004), with the average reduction was 14.8 ± 7.7% (95% confidence interval [CI]; from 9.3 to 20.3). It appears that 8 out of 10 participants (80.0%) receiving CN‐CRN intervention experienced mildly reduced kidney function (eGFR 60–89 ml/min/1.73 m^2^) as compared to 40.0% in CN group and 30.0% in CRM group. Nevertheless, no participant faced a clinically relevant reduction in eGFR (<60 ml/min/1.73 m^2^) throughout the course of the trial. The highest reduction in eGFR (23.9%) was noted in a participant (age 30, 69.5 kg) receiving CN‐CRN intervention.

**Table 1:** Changes in liver enzymes and estimated glomerular filtration rate (eGFR) during the study

	Baseline	At 5‐days follow‐up			*p* *
		CN	CN‐CRN	CRM	
Aspartate aminotransferase (IU/ml)	21.0 ± 2.6	20.1 ± 1.9	20.5 ± 3.2	20.2 ± 2.2	.940
Alanine aminotransferase (IU/ml)	18.2 ± 2.4	21.8 ± 6.6	21.4 ± 5.7	20.9 ± 3.4	.949
eGFR (ml/min/1.73 m^2^)	97.8 ± 8.3	89.8 ± 8.1	83.3 ± 10.6	91.7 ± 8.3	.004

Note

Values are mean ± *SD*.

* *p* value from two‐way mixed ANOVA (treatment vs. time interaction)

**Figure 4:**
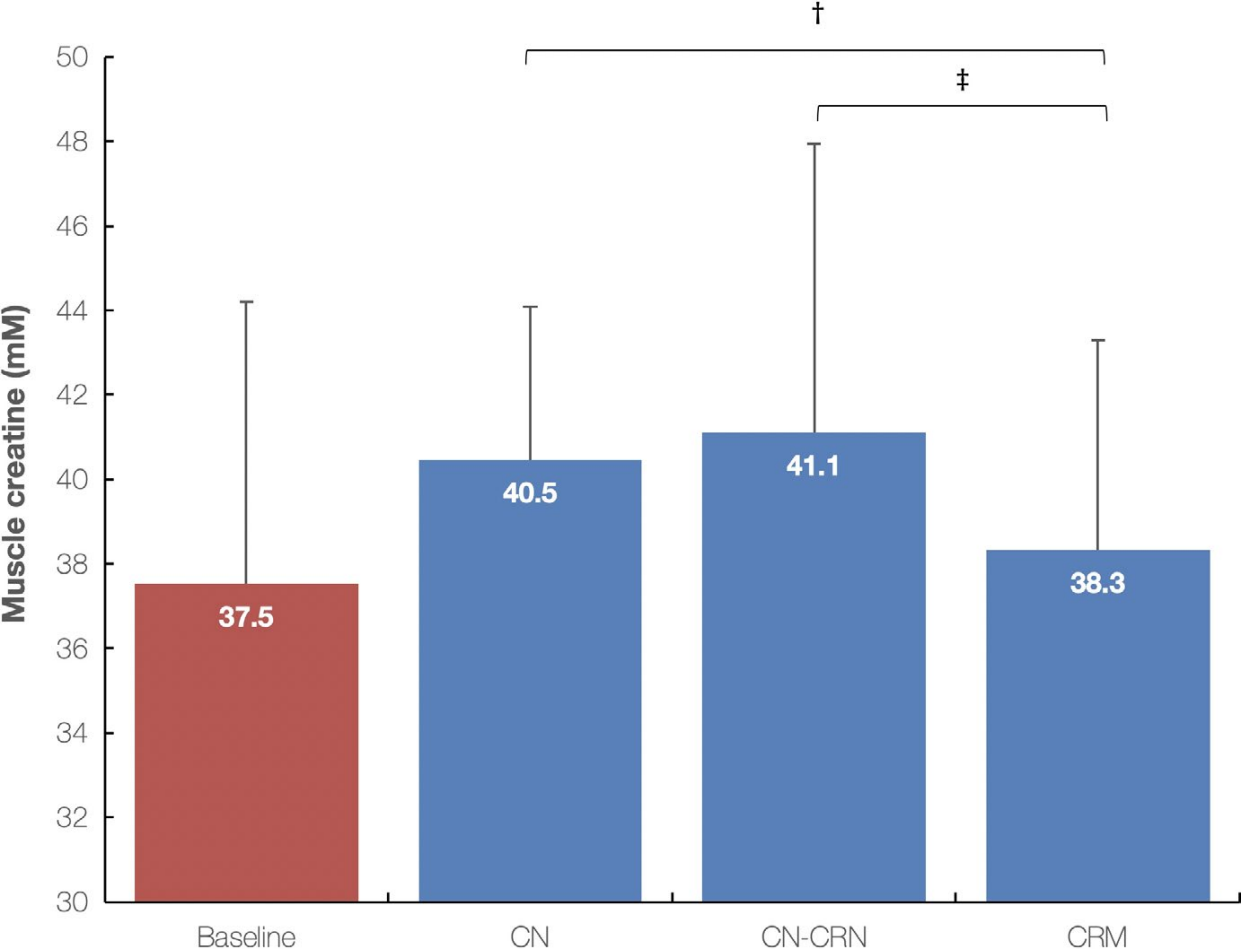
Total muscle creatine levels at baseline and 5 days postintervention for creatine nitrate (CN), creatine nitrate plus creatinine (CN‐CRN), and creatine monohydrate (CRM) trials. Values are mean ± *SD*. †Indicates significant difference for percent change in muscle creatine levels between CN and CRM trials (*p* = .01), and ‡Indicates significant difference for percent change in muscle creatine levels between CN‐CRN and CRM trials (*p* = .008)

**Figure 5:**
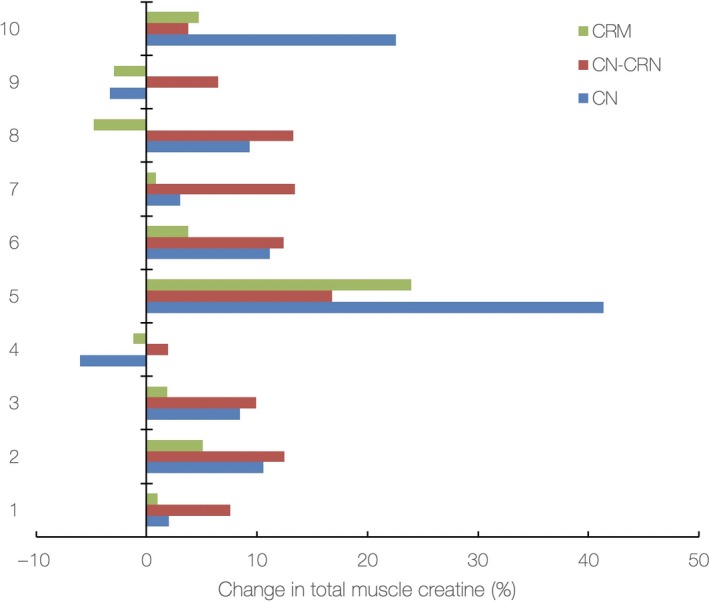
Individual changes (%) in total muscle creatine levels from baseline to 5‐day follow‐up for creatine nitrate (CN), creatine nitrate plus creatinine (CN‐CRN), and creatine monohydrate (CRM) trials

All three interventions induced a notable rise in muscle total creatine from baseline levels (37.5 ± 6.7 mM) to postadministration levels, with two‐way ANOVA with repeated measures revealed a significant effect of the intervention (*p* = .01), indicating different concentration changes in skeletal muscle creatine dependent on the intervention administered (Figure 4). It appears that CN‐CRN intervention caused the highest average increase in total muscle creatine levels (9.6%; 95% CI from 6.4 to 13.2), followed by CN (8.0%, 95% CI from 0.1 to 19.7) and CRM trials (2.1%, 95% CI from −2.5 to 8.9), with both CN‐CRN and CN were significantly superior to boost muscle creatine concentrations compared to CRM (*p* < .01). While 2 out of 10 participants were found to be nonresponsive to CN intervention (20.0%) (e.g., no amplification in muscle creatine levels found at follow‐up), and 3 participants out of 10 were nonresponsive in CRM trial (30%), no nonresponders were found after CN‐CRN intervention, with individual upswing in total muscle creatine varied from 2.0% (lowest increment) to 16.8% (highest increment) (Figure 5).

## 4 DISCUSSION

This pilot study demonstrated a superiority of CN‐CRN mixture to increase creatine bioavailability, serum creatine concentrations, and creatine distribution and absorption to the muscles in a cohort of young healthy men, as compared to traditional creatine products, such as pure CN and regular CRM. Although of somewhat unpleasant odor, CN‐CRN blend appeared relatively safe, with no major disturbances of safety biomarkers for liver and kidney function, and/or subjectively reported adverse events. This verification of advanced utilization of CN‐CRN mixture might affirm its future exploration and use as a novel supplemental formula in human nutrition.

### 4.1 CN‐CRN performance

Many innovative creatine supplements have been evaluated for effectiveness and safety during the past decade or so, with most products appeared inferior (or at least nonsuperior) in terms of utilization, bioavailability, and performance to gold‐standard CRM. For example, a buffered form of creatine was found less effective than CRM (Jagim et al., 2012) while several creatine analogs have shown significant side effects and limited applicability (Andres et al., 2017). Although CRM has been validated in many studies for its value and safety, its use seems to be somewhat limited due to several technical or performance constraints, including its solubility in water, stability, bioavailability, and/or performance in specific conditions (Alraddadi, Lillico, Vennerstrom, Lakowski, & Miller, 2018; Kieburtz et al., 2015). Here, we demonstrated that the addition of creatinine to supplemental creatine nitrate improves its utilization in the blood and skeletal muscle for both single‐dose and 5‐day interventions. A coadministration of creatine nitrate and creatinine has shown favorable serum profiles, with the mixture improves body exposure to creatine for up to 52.1% comparing to CRM for 2‐hr pharmacokinetics. This translates into the greater uptake of bioavailable creatine from the blood to the skeletal muscle, with CN‐CRN intervention (also CN) superiorly boosted muscle creatine levels as compared to CRM. The addition of creatinine to creatine nitrate perhaps changes chemical equilibrium in creatine‐to‐creatinine conversion in the blood, thus enhancing reverse reaction and an increase in serum creatine concentrations, and a perhaps higher saturation of cellular transporter for creatine (CRT1) that drives superior muscle consumption. Besides chemical equilibrium theory, CN‐CRN might positively affect creatine uptake by virtue of other mechanisms, including enhanced muscle blood flow through provision of additional nitrate (Richards et al., 2018), and favorable chemical environment (driven by nitrate group) that could optimize creatine–creatinine conversion (Jäger, Purpura, Shao, Inoue, & Kreider, 2011), that needs to be addressed in future studies. CRM appears to be absorbed substantially in the present study, which is in accordance with previous trials suggesting 98% absorption capability for creatine monohydrate (Jäger et al., 2011; Persky, Brazeau, & Hochhaus, 2003), yet two other formulations used here might have one of more advantages for blood and tissue uptake enlisted above.

The transformation of creatine to creatinine is a reversible nonenzymatic reaction, with chemical equilibrium may be shifted by different factors such as changes in temperature, pH or the concentration of a reactant or a product. For example, the elevation of temperature, as well as the lowering of the pH, favors the formation of creatinine while creatine is favored at high pH and low temperature (Wyss & Kaddurah‐Daouk, 2000). Specifically, changing the concentration of creatine or creatinine must follow Le Chatelier's principle, shifting the system in such a way to cancel the change to reach equilibrium again (Campbell, 1985). In our case, a boosted concentration of creatinine induced by exogenous intake perhaps moved a reaction backward, resulting in increased creatine and decreased creatinine concentrations. This has been confirmed for in vitro conditions, with creatinine may be hydrolyzed back to creatine in aqueous solutions, such as urine and blood (Lempert, 1959). On the other hand, an isotope‐labeled seminal study questioned reversibility of creatine to creatinine reaction for in vivo environment (Bloch & Schoenheimer, 1939), with most of the exogenous labeled creatinine was directly excreted into the urine while no significant exchange of the label with the body creatine was observed. Here, we provided a first indirect proof that creatinine might be hydrolyzed back to creatine in humans, as noted via favorable pharmacokinetics for serum creatine in a single‐dose CN‐CRN trial, although studies using isotope‐labeled creatinine are needed to address this.

### 4.2 Side effects and nonresponders

We reported no major adverse events after 5‐day supplementation with CN‐CRN mixture, besides a single‐case of nondiarrheal bowel movements in a sensible participant. CN‐CRN intervention affected no liver enzymes while eGFR seems to be moderately reduced (14.8%), while no participant experienced serum creatinine levels >120 µmol/L at 5‐day follow‐up. The safety profile after short‐term CN‐CRN supplementation thus appears to be acceptable, and perhaps similar to CRM (Kreider et al., 2017). Nevertheless, future trials with CN‐CRN mixture should cover a more extensive safety profile (including direct biomarkers of possible kidney stress), accounting also for possible mutagenicity and toxicity of exogenous creatinine (Wyss & Kaddurah‐Daouk, 2000). In addition, CN‐CRN appeared to favorably affect total muscle creatine concentrations in all recruited participants, while up to 30% of participants who received the other two interventions remained nonresponsive to creatine. The prevalence of nonresponders to creatine reported by previous studies was found to be ~25% (Kreider et al., 2017), which is in accordance with our results for both CN and CRM trials. However, an advantageous uptake of creatine by the skeletal muscle after creatine nitrate plus creatinine intervention, even in participants with a high initial level of muscle creatine (who are otherwise immune to creatine supplementation), perhaps nominates the mixture as highly applicable, particularly for individuals who are nonresponders to traditional creatine formulations.

### 4.3 Limitations and open questions

Although this study provided the first evidence about the performance of creatine–creatinine mixture in humans, few limitations have to be considered when study findings are interpreted. First, the duration of intervention appears to be comparatively short, thus preventing us to draw any firm conclusions about the medium‐ or long‐term efficacy and safety of creatine–creatinine supplementation. Second, the trial population included only young healthy men and was relatively small in size; it remains unknown how the intervention affects women, other age‐groups, or clinical populations, particularly those with kidney disorders. Third, we have not evaluated whether advanced tissue bioenergetics after intervention translates into physiological and clinical outcomes, including athletic performance. A change in the total amount of creatine cannot be directly extrapolated to a potential increase in performance, so additional performance studies are needed to evaluate this issue. Finally, other creatine–creatinine formulations (in addition to the 3‐to‐3 ratio used in the present trial) should be developed, along with a comparison with creatinine intervention alone, and their effects determined in well‐powered trials.

## 5 CONCLUSION

Short‐term supplementation with creatine nitrate plus creatinine appears to superiorly affect creatine levels in the circulation and skeletal muscle in comparison with either creatine nitrate or creatine monohydrate, thus advancing this experimental formulation as a preferred alternative to traditional creatine formulations. Nevertheless, the exact mechanism of creatine nitrate plus creatinine performance needs to be further elucidated, and more well‐designed long‐term studies in both athletic and clinical environment are required.

## CONFLICT OF INTEREST

The authors declare that they do not have any conflict of interest.

## ETHICAL APPROVAL

All experimental procedures were approved by the local IRB (Ethical Approval No. 102‐TL/2018).

## INFORMED CONSENT

Written informed consent was obtained from all study participants.
